# Liver, but Not Muscle, Has an Entrainable Metabolic Memory

**DOI:** 10.1371/journal.pone.0086164

**Published:** 2014-01-23

**Authors:** Sheng-Song Chen, Yolanda F. Otero, Kimberly X. Mulligan, Tammy M. Lundblad, Phillip E. Williams, Owen P. McGuinness

**Affiliations:** Department of Molecular Physiology and Biophysics, Vanderbilt University School of Medicine, Nashville, Tennessee, United States of America; University of Cordoba, Spain

## Abstract

Hyperglycemia in the hospitalized setting is common, especially in patients that receive nutritional support either continuously or intermittently. As the liver and muscle are the major sites of glucose disposal, we hypothesized their metabolic adaptations are sensitive to the pattern of nutrient delivery. Chronically catheterized, well-controlled depancreatized dogs were placed on one of three isocaloric diets: regular chow diet once daily (Chow) or a simple nutrient diet (ND) that was given either once daily (ND-4) or infused continuously (ND-C). Intraportal insulin was infused to maintain euglycemia. After 5 days net hepatic (NHGU) and muscle (MGU) glucose uptake and oxidation were assessed at euglycemia (120 mg/dl) and hyperglycemia (200 mg/dl) in the presence of basal insulin. While hyperglycemia increased both NHGU and MGU in Chow, NHGU was amplified in both groups receiving ND. The increase was associated with enhanced activation of glycogen synthase, glucose oxidation and suppression of pyruvate dehydrogenase kinase-4 (PDK-4). Accelerated glucose-dependent muscle glucose uptake was only evident with ND-C. This was associated with a decrease in PDK-4 expression and an increase in AMP-activated protein kinase (AMPK) phosphorylation. Interestingly, ND-C markedly increased hepatic FGF-21 expression. Thus, augmentation of carbohydrate disposal in the liver, as opposed to the muscle, is not dependent on the pattern of nutrient delivery.

## Introduction

In the clinical setting and specifically on the critical care setting, nutrient delivery, administered either via the enteral or parenteral route is commonly used. This nutritional support often causes hyperglycemia in hospitalized patients even if there is no history of diabetes [1]. Hyperglycemia is associated with higher morbidity and mortality [2]. The increased incidence is due to the underlying stress and inflammation combined with the need to deliver nutrition which has relatively high carbohydrate content. We observed the liver adapts to the carbohydrate diet that is infused continuously; it becomes an efficient consumer of glucose, which lowers the overall insulin requirements to sustain whole body glucose disposal, [3], [4]. This adaptation is independent of the route (enteral or parenteral) of nutrient delivery. Recent evidence suggests that restricting the pattern of nutrient delivery can alter gene expression of metabolically relevant enzymes in liver [5], [6]; the specific effect on hepatic glucose uptake and metabolism was not addressed. Muscle is also a major site of glucose disposal. One could speculate that, like the liver, the pattern of nutrient availability could alter the muscle's capacity to metabolize carbohydrate.

This study addressed two questions. First, does the adaptive increase in whole body glucose disposal seen after continuous nutrient delivery, persist if a normal feeding/fasting cycle is present? Second, are hepatic and muscle equally sensitive to the pattern of nutrient delivery? Using a chronically catheterized, depancreatized, conscious canine model, we demonstrate that in the setting of a controlled insulin environment, continuous nutrient delivery augments basal and glucose-stimulated hepatic and muscle glucose uptake in the absence of changes in insulin signaling. Moreover, when the nutrient delivery is restricted to the normal feeding pattern, which allows for a period of fasting, the liver retains the enhanced capacity to store and oxidize glucose, while the muscle loses this adaptation. These results suggest that the liver can be entrained to develop a “metabolic memory” that persists through a normal feeding/fasting cycle, while the muscle has no such memory.

## Methods

### Animal Husbandry

Male and female non-pregnant purpose bred mongrel dogs (n = 17), were fed standard Kal-Kan meat (Vernon, CA) and Purina Lab Canine Diet (Purina Mills, St. Louis, MO) once daily. The composition of the diet was 43% carbohydrate (62% non-protein calories), 31% protein and 26% fat. Dogs were housed in a facility that met Association for Assessment and Accreditation of Laboratory Animal Care International guidelines. All protocols were approved by the Vanderbilt University Medical Centre Institutional Animal Care and Use Committee.

### Animal Preparation

Under general anesthesia the pancreas was removed and blood sampling catheters were placed in the femoral artery, iliac vein and portal and hepatic vein. Infusion catheters were placed in the portal vein via the splenic vein so that insulin can be delivered by its physiological route. In the group fed once a day (ND-4), a duodenal catheter was placed. The group with continuous nutrient delivery (ND-C) a catheter was placed in the inferior vena cava (IVC). Flow probes (Transonic Systems, NY) were placed on the hepatic artery, portal vein and external iliac artery [7]. Prior work showed that the hepatic response to enteral and parenteral nutrient delivery was similar [3], [4]. Free ends of the catheters, and flow probes were exteriorized and placed in subcutaneous pockets [8]. After removal of the pancreas, dogs were treated with subcutaneous injections of regular (Eli Lilly, Indianapolis, IN) and NPH (intermediate-acting) insulin daily until a continuous infusion of nutrition was initiated. Pancrease was added to the diet to facilitate digestion.

### Experimental design and nutrient delivery

**After surgery** (≥14 days) free catheter ends were exteriorized from the subcutaneous pocket under local anesthesia (2% Lidocaine; Abbott, Chicago, IL). Dogs wore a jacket (Alice King Chatham, Los Angeles, CA) to hold the nutrition bag and pumps and either received chow once a day (Chow; n = 6), or were placed on nutrient delivery for 4 days (ND). Animals were trained to eat the entire chow meal within 60 min. The ND group was divided in two different groups depending on the feeding pattern: infusion over a 4 h period once a day through the duodenal catheter (ND-4; n = 6) or continuous infusion via intravenous route (ND-C; n = 5) using an ambulatory infusion pump (Dakmed, Buffalo, NY). Nutritional support was composed of elemental nutrients, which can rapidly be absorbed. In contrast chow is slowly absorbed in canines [9]. Thus to better match the availability of nutrient in the ND-4 and Chow group, the ND-4 was infused enterally over a 4 h period. A variable intraportal infusion of insulin (Walkmed-350; Lakewood, CO), was given to maintain euglycemia for the entire 4 days. In the ND-C, during the first 48 h, the insulin infusion was variable to maintain euglycemia (∼120 mg/dl) and was thereafter held constant at 0.4 mU·kg^−1^·min^−1^. In the other two groups, when food was administered, the insulin infusion rate (1.5–2.0 mU·kg^−1^·min^−1^) was increased to prevent the glucose from increasing above 180 mg/dl and then gradually tapered to a basal rate (∼0.25 mU·kg^−1^·min^−1^) over a 6–8 hr period, after which the insulin infusion rate was held at the basal insulin rate until the next feeding period.

Dogs received either chow or a liquid diet as the sole exogenous caloric source for 4 days. The ND and Chow were isocaloric based on predicted resting energy expenditure. The ND included glucose (Dextrose or Polycose for enteral nutrition; 75% of non-protein calories), lipids (20% Intralipid®; Baxter Healthcare, Deerfield, IL; 25% of non-protein calories), amino acids (Travasol®), saline, potassium phosphate, and a multivitamin supplement.

### Experimental Protocol

On the morning of day 5, the free ends of the catheters and flow probes were exteriorized and the dog was placed in a Pavlov harness. Regardless the previous treatment, all groups were infused intravenously with Travasol and Intralipid at an identical rate as the ND-C group received during the past 4 days, throughout the study period. Insulin was infused at 0.4 mU·kg^−1^·min^−1^ for the duration of the study. The glucose concentration and insulin infusion rates are designed to match the concentrations of insulin and glucose seen in canines that have an intact pancreas and are receiving chronic nutrient delivery [3], [4]. Glucose infusion rate was adjusted until glycemia was clamped at 120 mg/dl. All animals then received a primed (10 µCi) continuous (0.1 µCi/min) infusion of [3-^3^H] glucose and [U-^14^C] glucose (0.05 µCi/min) into the inferior vena cava catheter for the duration of the study. After a tracer equilibration period (0 to 90 min), organ substrate balance was assessed between 90–120 min. To assess the ability of both the liver and muscle to enhance glucose uptake, the glucose concentration was then increased to 220 mg/dl and substrate balance was assessed between 210 and 240 min. Blood samples were taken from the artery, portal vein, hepatic vein, and iliac vein. At the conclusion of the study period, animals were sacrificed with a lethal dose of pentobarbital sodium. Liver and muscle biopsies were taken and frozen with a Wallenburg clamp pre-cooled in liquid nitrogen and stored at −80°C.

### Calculations

Net hepatic and muscle substrate (glucose, lactate, alanine, and glycerol β-hydroxybutyrate (β-OHB)) uptake and fractional extraction were calculated using arterial-venous difference techniques as previously described [3], [4]. Plasma glucose concentration was assessed using an Analox Glucose Analyzer (GM-9; Lunenburg, MA). Blood lactate, alanine, β-hydroxybutyrate (β-OHB) and glycerol were assessed using fluorometric enzymatic analysis [10]. The concentration of nonesterified fatty acids (NEFA) was determined spectrophotometrically (Wako Chemicals, Richmond, VA). Hepatic glucose oxidation was calculated as the net production of ^14^CO_2_ by the liver divided by the inflowing ^14^C glucose specific activity (ratio of hepatic [^14^C] glucose load and hepatic glucose load). Net non-hepatic glucose uptake was calculated as the difference between exogenous glucose infusion rate (GIR) and NHGU. Net non-hepatic carbohydrate disposal was calculated as the sum of net non-hepatic glucose uptake and net hepatic lactate release.

### Tissue analysis

Protein from liver and muscle was extracted with a homogenization buffer (50 mM Tris, 1 mM EDTA, 1 mM EGTA, 10% glycerol, 1% Triton X-100 at pH 7.5,1 mM DTT, 1 mM PMSF, 5 ug/mL protease inhibitor cocktail, 10 ug/mL trypsin inhibitor, 50 mM NaF, and 5 mM NaPP) and stored at −80°C until used. SDS-PAGE was carried out with 30 µg of protein and transferred to a polyvinylidene fluoride membrane as previously described [11]. Western blot was done with anti-glucokinase (GCK) antibody (generously donated by Dr. Masakazu Shiota); anti-glucokinase regulatory protein (GCKRP) antibody (sc-11416 Santa Cruz Biotechnology, CA), anti-phospho-AKT (pAkt) Ser 743 antibody (9271), anti-Akt antibody (9272), anti-phospho-AMP-activated protein kinase (pAMPK) Thr 172 antibody (2531), anti-AMPK antibody (2532). As loading control anti-β-actin antibody (4967) was used (all antibodies obtained from Cell Signalling, MA, unless specified otherwise). Membranes were developed using enhanced chemiluminescence and images were quantified with ImageJ software.

Total mRNA was extracted from liver and muscle using the Trizol® protocol (Invitrogen, CA) and the RNeasy fibrous tissue kit (Qiagen Sciences, MD). cDNA was synthesized from 2 µg mRNA with the High Capacity cDNA transcription kit (Applied Biosystems, CA). PCR amplification reactions were performed in duplicate using the Bio-Rad detection system. Gene expression was normalized to beta-actin (ACTB) levels. PCR amplification to detect GCK, GCKRP, PDK-4, FGF-21 and the housekeeping ACTB was performed with TaqMan® gene expression assays (Applied Biosystems). The amount of target, normalized to endogenous gene and relative to the Chow is given by RQ = 2^−ΔΔCt^, [ΔCt = Ct (target gene)−Ct (ACTB); ΔΔCt = ΔCt for any sample−ΔCt for the control].

Liver and muscle glycogen [12] and hepatic glycogen synthase (GS) enzymatic activity [13] were assessed.

### Statistics

All data were expressed as means ± SEM. Statistical analysis was performed using mixed effects model for repeated measures. Topelitz covariance was used in the model. Pair wise comparisons were not made unless the overall model for each variable tested met the significance level of p<0.05. All data are reported as an average of 5 points during the sampling period. Paired comparisons were performed with the two-tailed Student's *t* test. Sigma Plot 12.0 (Aspire Software International) was used for all statistical analysis.

## Results

### ND increased hepatic glucose uptake, oxidation, and glycogen synthesis

**Figure 1:**
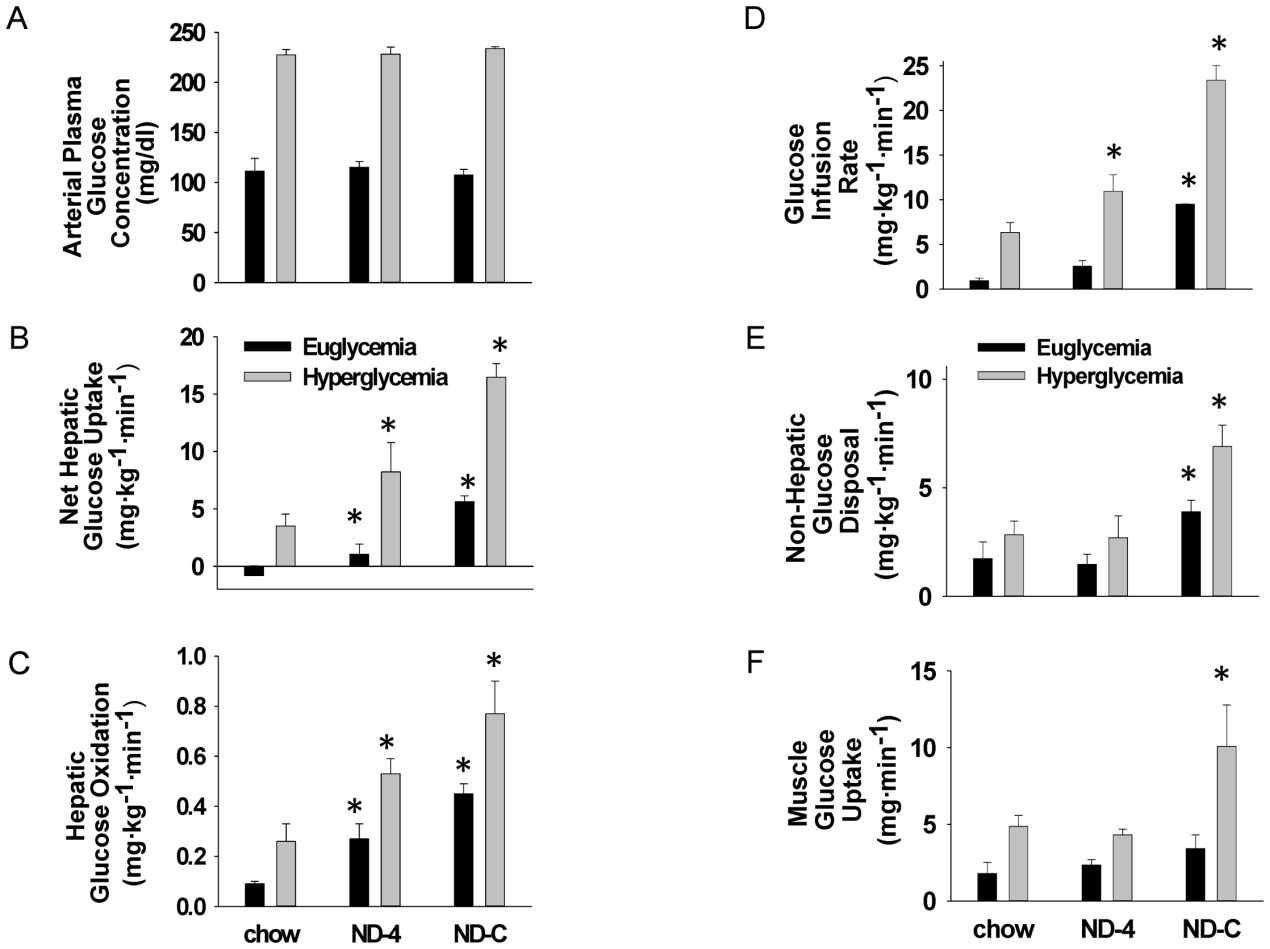
Arterial plasma glucose concentration, net hepatic glucose uptake and hepatic glucose oxidation, exogenous glucose infusion rate, net non-hepatic glucose uptake and net muscle glucose uptake in chronically catheterized conscious insulin-treated depancreatized dogs in the presence of basal intraportal insulin at euglycemia and hyperglycemia. Animals were either on a chow diet or fed isocaloric nutritional support as a constant infusion (ND-C) or once a day for 4 days (ND-4). * indicate significantly different from chow (p<0.05).

In all groups (regardless of the sex) arterial glucose and insulin concentrations during the euglycemic and hyperglycemic periods were matched (Fig. 1A and Table 1). Hepatic artery (7.4±2.1, 4.7±0.7 and 5.2±0.8 ml·kg^−1^·min^−1^; Chow, ND-4, ND-C, respectively) and portal vein (23.4±2.1, 22.6±2.6 and 21.5±1.6 ml·kg^−1^·min^−1^) blood flow were not altered by nutrient delivery. Liver weights (g/kg) were similar (34±2, 40±6, 37±6).

**Table 1:** Arterial plasma insulin and glucagon in chronically catheterized conscious insulin-treated depancreatized dogs on a chow diet or fed an isocaloric nutritional support as a constant infusion (ND-C) or once a day for 4 days (ND-4).

	Chow		ND-4		ND-C	
Glucose concentration (mg/dl)	120	220	120	220	120	220
Insulin (µU/ml)	6.2±0.5	7.1±0.6	7.9±1.5	8.2±1.4	7.4±0.5	8.6±0.8
Glucagon (pg/ml)	36±7	27±4	48±7	37±7	19±2*	18±4*

On the day of the study in the presence of intraportal insulin (0.4 mU·kg^−1^·min^−1^) glucose was clamped at euglycemia (120 mg/dl) and then hyperglycemia (220 mg/dl).

* P<0.05 vs. Chow.

At euglycemia (120 mg/dl), NHGU increased in ND-4 and increased further in ND-C relative to Chow (Figure 1B and C). In response to hyperglycemia (220 mg/dl), glucose-stimulated NHGU was increased in ND-4 and ND-C relative to Chow. In parallel with the increase in NHGU, hepatic glucose oxidation and the glucose infusion rate required to match glucose concentrations were also increased (Fig. 1 C and D). Peripheral (non-hepatic) glucose disposal and muscle uptake were increased in ND-C compared to ND-4 and Chow (Figure 1E, F).

### ND increased hepatic glycogen and lactate metabolism

**Figure 2:**
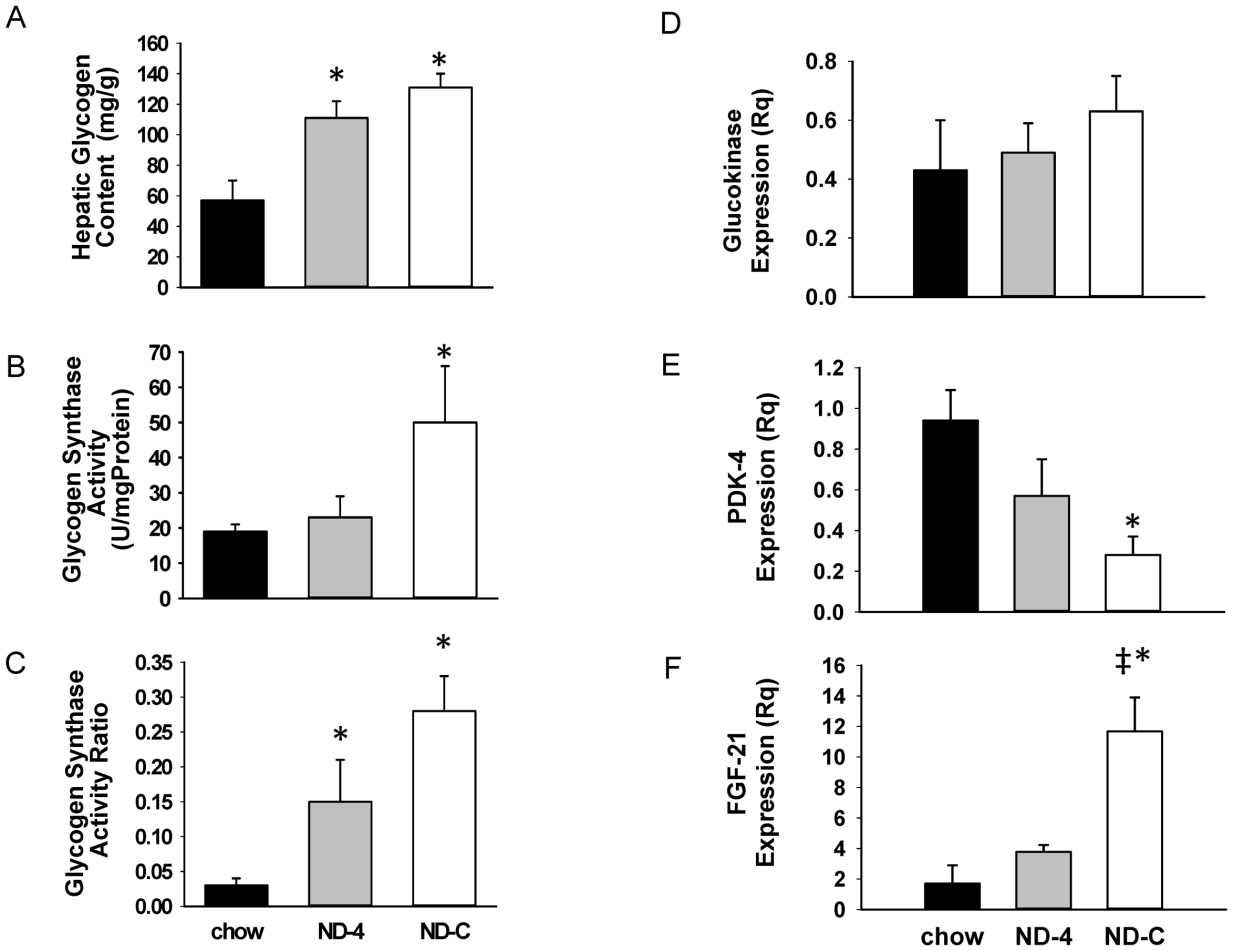
Glycogen content, glycogen synthase activity and activity ratio and the expression of GK, PDK4 and FGF-21 in the liver from chronically catheterized conscious insulin- treated depancreatized dogs that were either on a chow diet or fed isocaloric nutritional support as a constant infusion (ND-C) or once a day for 4 days (ND-4). * and ‡ indicate significantly different from chow and ND-4, respectively (p<0.05).

Despite equivalent caloric intake in all groups, in ND-C and ND-4, glycogen content was increased more than 2-fold in Chow (Figure 2A) and the liver was a net producer of lactate (Table 2). In Chow, the liver was a net consumer of lactate initially, but then switched to lactate production in response to hyperglycemia, which accounted for 19% of the NHGU. Despite elevated arterial lactate in ND-4, hepatic lactate release exceeded NHGU, implying net glycogenolysis (Table 2). In response to hyperglycemia, lactate release increased but to a lesser extent than the increase in liver glucose uptake. Thus the lactate release that could be accounted for by NHGU was 20%. In ND-C, the liver was a marked producer of lactate, accounting for 30% of NHGU at euglycemia. In response to hyperglycemia, lactate release did not increase further despite a marked increase in NHGU. In all groups the increase in glucose-dependent NHGU was accompanied by an increase in tracer determined glycogen synthesis, with ND-C having the largest increase (0.4±0.1, 0.6±0.2 and 2.2±0.6 mg·kgBW^·1^·min^−1^ in Chow, ND-4 and ND-C,).

**Table 2:** Arterial Blood concentration (µM), net hepatic uptake (or release; µmol·kg^−1^·min^−1^) and fractional extraction of lactate, alanine, glycerol, non-esterified fatty acids (NEFA) and β-OHB in chronically catheterized conscious insulin-treated depancreatized dogs on a chow diet or fed an isocaloric nutritional support as a constant infusion (ND-C) or once a day for 4 days (ND-4).

	Chow		ND-4		ND-C	
Glucose Concentration (mg/dl)	120	220	120	220	120	220
**Lactate**						
Concentration	691±162	1359±267	3340±655*	4031±768*	1090±199	1490±260
Net Hepatic Release	−1.8±2.8	6.0±6.3	19.5±2.4*	33.7±11.7*	33.7±2.9*	34.3±6.4*
**Alanine**						
Concentration	629±112	719±101	1980±130*	1888±74*	790±122	889±164
Net Hepatic Uptake	3.9±0.6	4.2±2.0	4.1±1.2	2.5±1.6	0.9±0.6*	1.8±0.6*
Net hepatic Fractional Extraction	0.22±0.03	0.19±0.08	0.07±0.02*	0.05±0.03*	0.05±0.03*	0.09±0.04*
**Glycerol**						
Concentration	88±18	70±9	87±19	69±13	48±6*	50±6*
Net Hepatic Uptake	1.8±0.3	1.6±0.3	1.7±0.2	1.2±0.1	0.9±0.1*	0.9±0.1*
Net Hepatic Fractional Extraction	0.74±0.04	0.78±0.06	0.68±0.01	0.69±0.02	0.73±0.05	0.72±0.07
**NEFA**						
Concentration	688±189	453±52	544±48	372±22	209±17*	175±23*
Net Hepatic Uptake	3.2±0.6	2.5±0.3	1.3±0.9	0.8±0.7	0.5±0.2*	0.5±0.1*
Net hepatic Fractional Extraction	0.26±0.07	0.28±0.06	0.12±0.07	0.09±0.09	0.14±0.05	0.14±0.03
**β-OHB**						
Concentration	42±13	27±3	93±16	72±24	20±1*	21±3*
Net Hepatic Release	1.1±0.3	0.5±0.1	1.0±0.3	0.7±0.2	0.4±0.1*	0.3±0.1*

On the day of the study in the presence of intraportal insulin (0.4 mU·kg^−1^·min^−1^) glucose was clamped at euglycemia (120 mg/dl) and then hyperglycemia (220 mg/dl).

* P<0.05 vs. Chow.

### ND increased hepatic GS and pyruvate oxidation capacity independent of insulin or AMPK signaling

**Figure 3:**
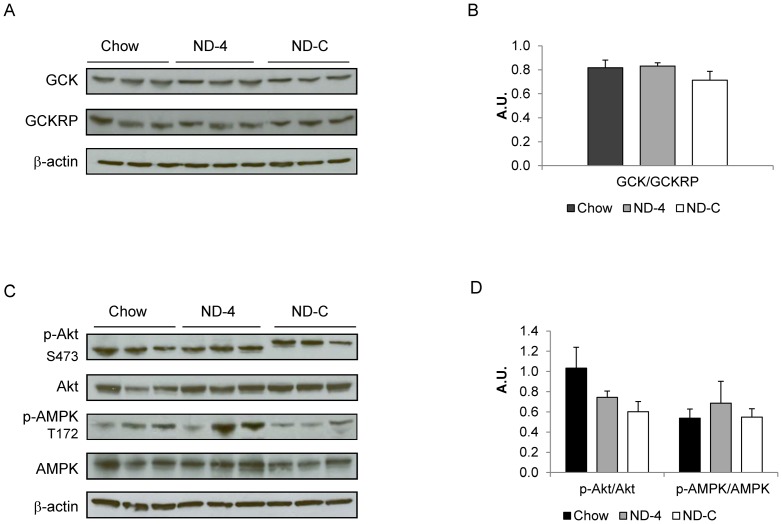
Protein expression and ratio of GCK and GCKRP (A, B) and activation of AMPK and Akt (C,D) in the liver from chronically catheterized conscious insulin treated depancreatized dogs that were either on a chow diet or fed isocaloric nutritional support as a constant infusion (ND-C) or once a day for 4 days (ND-4). Western blot images are representative of three experiments. Ratios are calculated as the average of phosphorylated protein divided by total amount of protein and represented in arbitrary units (A.U.).

Consistent with the increase in hepatic glycogen synthesis, the GS activity ratio was augmented in the ND groups, and total GS activity was increased as well in ND-C (Figure 2B, C). The increase in NHGU could not be explained by an increase in glucokinase mRNA (Figure 2D) or protein (Figure 3 A, B) expression. A decrease in PDK-4 mRNA expression in ND-C (Figure 2E) is consistent with the accompanying increase in hepatic pyruvate (i.e. glucose) oxidation. ND-C and ND-4 did not alter Akt or AMPK activation (Figure 3 C, D). Interestingly, the increase in pyruvate oxidation occurred despite a marked increase in FGF-21 expression (Figure 2F). The increase occurred despite a lower level of glucagon, a known stimulator of FGF-21 expression, in ND-C (Table 1).

### Continuous ND decreased hepatic fat and alanine metabolism

ND-C decreased hepatic alanine uptake and fractional extraction relative to chow and ND-4 (Table 2). Arterial NEFA and glycerol and hepatic uptake of glycerol and NEFA were decreased in ND-C, indicating a decrease in lipolysis. This decrease in NEFA uptake was paralleled by a decrease in hepatic fat oxidation (i.e. hepatic β-OHB production).

### Muscle glucose uptake and lactate disposal are only enhanced by continuous ND

Glucose uptake by non-hepatic tissues (non-hepatic glucose uptake = glucose infusion rate-NHGU), and specifically skeletal muscle, was enhanced by ND-C (Figure 1E, F) both in the presence of euglycemia and hyperglycemia. This was absent in ND-4. Interestingly, despite receiving the identical diet as ND-C, glucose-dependent muscle glucose uptake was not increased in ND-4 relative to chow (Fig. 1E). Muscle (iliac artery) blood flow tended (p = 0.08 vs. Chow and p = 0.09 vs. ND-4) to increase with continuous ND (91±7, 107±16 and 143±20 ml/min; chow, ND-4 and ND-C). In both ND groups, muscle was a net lactate consumer. The uptake and fractional extraction of lactate by muscle was markedly increased in ND-C, which is consistent with an augmentation of muscle pyruvate oxidation. In ND-4 the increase was primarily facilitated by the elevated lactate delivery to the muscle, as net fractional extraction was not increased (Table 3). While hyperglycemia increased muscle lactate uptake in Chow and ND-4 groups, it did not increase further in ND-C.

**Table 3:** Muscle lactate load, uptake and fractional extraction in chronically catheterized conscious insulin-treated depancreatized dogs on a chow diet or fed an isocaloric nutritional support as a constant infusion (ND-C) or once a day for 4 days (ND-4).

	Chow		ND-4		ND-C	
Glucose Concentration (mg/dl)	120	220	120	220	120	220
**Lactate**						
Net Muscle Load (µmol·min^−1^)	63±11	154±34	345±107*	410±128*	119±41	190±69
Net Muscle Uptake (µmol·min^−1^)	−0.6±3.0	18.8±6.3	31±21*	65±35*	35±10*	49±11
Net Muscle Fractional Extraction	−0.02±0.06	0.12±0.03	0.10±0.05	0.15±0.06	0.35±0.11*	0.24±0.05

On the day of the study in the presence of intraportal insulin (0.4 mU·kg^−1^·min^−1^) glucose was clamped at euglycemia (120 mg/dl) and then hyperglycemia (220 mg/dl).

* P<0.05 vs. Chow.

### ND-C increased muscle pyruvate oxidation and AMPK signaling but not insulin signaling

**Figure 4:**
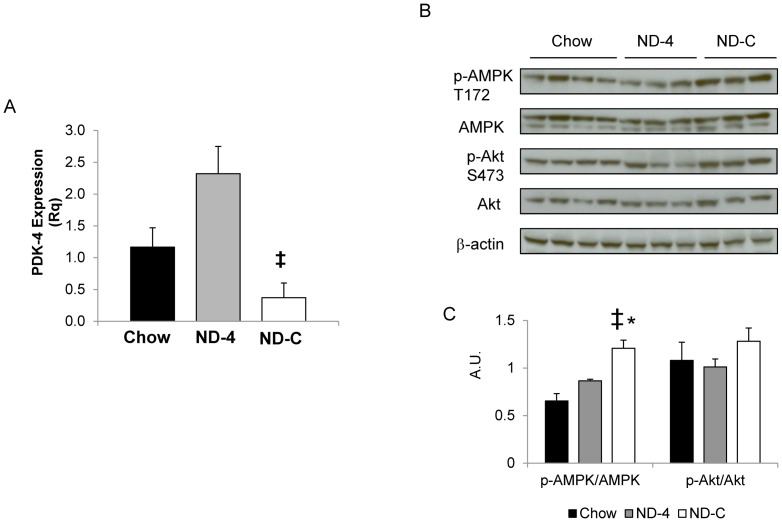
PDK4 mRNA expression (A) and activation ratio of AMPK and Akt (B) in the muscle from chronically catheterized conscious insulin treated depancreatized dogs that were either on a chow diet or fed isocaloric nutritional support as a constant infusion (ND-C) or once a day for 4 days (ND-4). mRNA expression is represented as Rq (relative expression to endogenous and control group). Western blot images are representative of three experiments. Ratios are calculated as the average of phosphorylated protein divided by total amount of protein and represented in arbitrary units (A.U.). * and ‡ indicate significantly different from chow and ND-4, respectively (p<0.05).

In parallel with the increase in muscle glucose uptake and enhanced ability of the muscle to metabolize lactate, the expression of PDK-4 was decreased in ND-C relative to chow (Figure 4A). This was paralleled by increased phosphorylation of AMPK in the absence of changes in insulin signaling (Akt phosphorylation). In ND-4, the diminished capacity of muscle to take up glucose and remove lactate was associated with a failure to decrease PDK-4 expression and activate AMPK. Muscle glycogen content was not increased in ND-4 or ND-C as compared to Chow (5.8±2.1, 7.2±1.9 and 8.1±2.2 mg/g; chow, ND-4 and ND-C).

## Discussion

Hyperglycemia in response to nutritional support is common in the hospitalized setting. Prevention of hyperglycemia in response to the relatively glucose rich nutrition requires adaptations in the liver and muscle to enhance the capacity to both store and oxidize glucose. We used chronically catheterized depancreatized dogs with controlled insulin replacement to assess the impact of the pattern of nutrient delivery in the absence of changes in daily caloric intake on basal and glucose stimulated hepatic and muscle glucose metabolism. Delivery of glucose-rich nutrition amplified the ability of the liver to oxidize and store glucose, irrespective of the pattern of nutrient delivery. In contrast, muscle augmented its capacity to oxidize and store glucose only when the nutrients were delivered continuously. When the pattern of nutritional support followed the normal feeding pattern (ND-4), the muscle adaptation was absent. Thus, the liver maintains a metabolic memory of changes in nutrients that is relatively insensitive to the pattern of nutrient delivery, while muscle lacks that memory.

Compared to chow feeding, nutritional support augmented basal liver glucose uptake and lactate release. This is consistent with our prior work. The adaptive response is not dependent on the route of nutrient delivery; continuous enteral or parenteral nutrient delivery augments liver glucose uptake and lactate release [3], [4]. What is surprising is that when the nutrients were only given once a day, the liver retained a significantly increased capacity to take up and store glucose. Liver glycogen content was much higher in animals receiving the glucose enriched diet. This increase occurred despite both the chow and ND-4 groups having the same chronic insulin and glucose environment. Recent data suggest that increased glycogen content impairs rather than augments liver glucose uptake [14]. However, a caveat to that study was that the glycogen content was augmented by the infusion of fructose, which activated AMPK and impaired insulin signaling in the liver [14]. AMPK was not activated by ND-4 in our study. Moreover, in our prior work, hepatic glycogen content did not alter the hepatic adaptation to nutrient delivery [15].

Nutrient delivery enhanced the glycogen synthetic capacity of the liver. An increase in glucose-dependent liver glycogen synthesis could be due to an increase in GCK and/or GS activity. However, neither the expression nor the protein content of GCK, nor its allosteric inhibitor GCKRP, was altered. Rather the primary effect was an enhancement of glucose-6-phosphate dependent activation of GS. With continuous nutrient delivery, total GS activity was increased as well. This is consistent with the observed increase in glucose-dependent glucose uptake with no alteration in insulin signaling. Genetic defects that limit hepatic glycogen synthetic capacity are known to impair glucose tolerance in mice and humans [16], [17]. The mechanism for the greater allosteric activation of GS is unclear. Neither GS kinase content nor GS phosphorylation was altered (data not presented). While insulin would be expected to augment GCK expression, in this study we controlled the insulin environment both acutely and chronically. This may explain why we did not observe an alteration in hepatic GCK. It would also suggest that the pattern of nutrient delivery combined with the availability of glucose plays a major role in determining the augmented glycogen synthetic capacity of the liver.

In addition to augmenting glycogen synthesis, the nutrient delivery augmented hepatic glycolytic flux and pyruvate oxidation. When the glucose rich nutrition was either given continuously or only once a day, hepatic lactate release was elevated both in the presence of euglycemia and hyperglycemia. The expression of PDK-4, an inhibitor of pyruvate oxidation, was decreased as hepatic glucose oxidation increased. In addition, a reciprocal decrease in free fatty acid uptake and hepatic fat oxidation (decreased β-OHB release) by the liver was observed, which likely contributed to the increase in pyruvate flux in the liver. In contrast to ND-C, where the majority of the lactate released by the liver was derived from plasma glucose, in ND-4 hepatic glycogen breakdown supplied some of the lactate carbon, as it was not a consumer of glucose at euglycemia. However when the plasma glucose was increased to 220 mg/dl, glycogenolysis was suppressed, and the majority of the lactate released by the liver was derived from plasma glucose. This suggests that insulin was unable to restrain the breakdown of liver glycogen to the same extent in the Chow and both ND groups; however the liver still retained the metabolic memory to augment glucose oxidation in response to hyperglycemia. In the Chow group, the lower hepatic glycogen content may in part explain the lower rate of glycogenolysis. However, in the ND-C group, while glycogen content was high, we found no evidence of persistent net hepatic glycogen breakdown. The glucagon concentration was lower in the continuous group. Moreover, net hepatic fractional extraction of alanine by the liver, a very sensitive marker of liver glucagon tone [18], was also decreased. Thus, it is possible the small increase in glucagon is able to modulate hepatic metabolism [7].

Surprisingly, the adaptation of the muscle to ND was very sensitive to the pattern of delivery. Despite the benefits afforded to the liver by the high glucose diet, this benefit did not extend to the muscle if the animal was allowed to follow its normal feeding/fasting cycle. Relative to both the Chow and ND-4, muscle glucose uptake and fractional extraction were strikingly increased with continuous ND, especially in the response to hyperglycemia. This would suggest that the transition to a physiologic fast markedly limited the capacity of the muscle to store and oxidize carbohydrate. Muscle glycogen content can both augment and inhibit insulin-mediated glucose uptake in skeletal muscle [19], [20]; however, muscle glycogen content was not altered. While muscle lactate fractional extraction was decreased in ND-4, muscle lactate uptake and thus muscle carbohydrate uptake was sustained. This was due to accompanying elevations in plasma lactate concentration, which was sustained by hepatic release of lactate. Pyruvate oxidation decreases as humans transition into a fast [21]. As PDK-4 expression increased in the ND-4 versus ND-C, alterations in pyruvate dehydrogenase activity likely determine carbohydrate and pyruvate oxidation. Prior work has suggested high carbohydrate diets decrease PDK-4 [22]. As ND-C also activated AMPK, this may have amplified muscle glucose uptake and glycogen synthesis [23]. Given the very high lactate clearance in the ND-C versus ND-4, muscle pyruvate oxidation was likely augmented. In response to hyperglycemia, muscle glucose uptake increased without an accompanying increase in lactate release, suggesting the adaptation augments both glycogen synthesis and pyruvate oxidation [24]. Muscle carbohydrate disposal is highest in the fed state. As most investigators fast their subjects, this allows the muscle to de-adapt, which could hide the impact of dietary manipulations on muscle metabolism. The fasted metabolic state may not reflect the true impact of the diet on muscle metabolism.

A number of other factors may have contributed to the absence of the muscle adaptive response in ND-4. The failure to augment muscle glucose uptake was accompanied by higher free fatty acid concentrations in ND-4 versus ND-C in the face of equivalent insulin concentrations. The failure to completely suppress glucagon may have contributed to the persistent lipolysis in the ND-4 [7]. In ND-4 nutrition was given via the enteral route. Enteral glucose delivery is known to inhibit muscle glucose uptake by activating the portal glucose signal; this is rapidly reversible when the portal signal is removed [25]. It is unlikely to explain the decrease in muscle glucose disposal, however, as the animals were not receiving enteral glucose when the response of the muscle to hyperglycemia was assessed. Interestingly, muscle blood flow, a known modulator of muscle glucose uptake [26], tended to decrease in ND-4 relative to ND-C.

Surprisingly, continuous ND markedly increased hepatic expression of FGF-21, which was absent if a normal feeding-fasting cycle was allowed. FGF-21 is thought to be primarily a fasting hormone whose expression is facilitated by glucagon [27]. However, FGF-21 was increased in the continuously fed state, where glucagon tone is low. While we were unable to measure the plasma concentration of canine FGF-21, this is consistent with reports that glucose can activate FGF-21 expression and secretion in hepatocytes [28], [29]. Consistent with our *in vivo* data, FGF-21 increases glucose uptake in human myotubes and muscle in absence of changes in insulin signaling [30] and activates AMPK at least in adipocytes [31]. The elevated hepatic expression of FGF-21 may have increased circulating levels of such a factor which could contribute to the enhancement in muscle glucose uptake (AMPK activation). In the liver, FGF-21 may facilitate changes in hepatic glycolytic flux (by suppressing gluconeogenesis) [28], [32], [33], [34] independent of changes in insulin signaling [35]. Obesity and overfeeding increase FGF-21; it has been proposed that obesity causes FGF-21 resistance [36]. However, the sustained availability of nutrients and insulin that is seen in obesity may be facilitating FGF-21 expression.

This study contrasts with work suggesting that restricting feeding to shorter periods than *ad libitum* in high fat fed mice has a metabolic benefit [6]. In our studies, animals were on an isocaloric diet for 5 days. Thus it is possible that in settings of caloric excess, restricted feeding patterns may have some offsetting effects that allow for the disposition of excess calories. It is also possible that some of the adaptations would change if we looked at a much longer duration of nutrient infusion. In those studies the ability of muscle and/or liver to dispose of carbohydrate was not evaluated. When glucose tolerance was evaluated, it was in the fasting, glycogen-depleted state which would hide any adaptive response in muscle and possibly liver.

**Figure 5:**
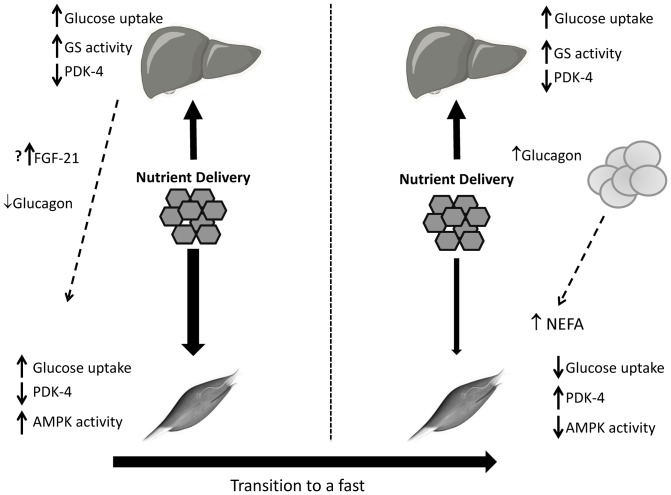
Schema representing the hepatic and muscle response to sustained nutrient delivery and the response to a physiologic fast. In response to sustained glucose availability the capacity of both the liver and muscle to oxidize and store carbohydrate is augmented. This is accompanied by low rates of lipolysis, glucagon secretion and augmented FGF-21. In response to a physiologic overnight fast glucagon and lipolysis increase and FGF-21 decreases which suppress the capacity of the muscle to oxidize and store carbohydrate, while having limited effects on the capacity of the liver to store and oxidize glucose.

In conclusion, in response to nutrient delivery, hepatic and muscle carbohydrate-dependent glucose disposal are augmented (Figure 5). This increase allows for the efficient storage and oxidation of exogenous glucose. Interestingly, liver and muscle have a differential sensitivity to the pattern of nutrient delivery. The liver retains the metabolic memory to store and metabolize carbohydrate even if a normal feeding/fasting cycle occurs. In contrast, this memory is rapidly lost in muscle. This may be facilitated by a concomitant rise in glucagon and lipolysis and a fall in FGF-21. Thus, dietary patterns that sustain carbohydrate availability may be able to take advantage of this combined adaptation in liver and muscle to facilitate efficient carbohydrate disposal and minimize hyperglycemia in the hospitalized setting.
